# Design and methods of Shape Up Under 5: Integration of systems science and community-engaged research to prevent early childhood obesity

**DOI:** 10.1371/journal.pone.0220169

**Published:** 2019-08-01

**Authors:** Julia M. Appel, Karen Fullerton, Erin Hennessy, Ariella R. Korn, Alison Tovar, Steven Allender, Peter S. Hovmand, Matt Kasman, Boyd A. Swinburn, Ross A. Hammond, Christina D. Economos

**Affiliations:** 1 Friedman School of Nutrition Science and Policy, Tufts University, Boston, Massachusetts, United States of America; 2 Department of Nutrition and Food Sciences, University of Rhode Island, South Kingston, Rhode Island, United States of America; 3 Global Obesity Centre (GLOBE), Centre for Population Health Research, Deakin University, Geelong, Australia; 4 Social System Design Lab, George Warren Brown School of Social Work, Washington University, Saint Louis, Missouri, United States of America; 5 Center on Social Dynamics and Policy, The Brookings Institution, Washington D.C, United States of America; 6 School of Population Health, University of Auckland, Aukland, New Zealand; University of Botswana Faculty of Medicine, BOTSWANA

## Abstract

Shape Up Under 5 (SUU5) was a two-year early childhood obesity prevention pilot study in Somerville, Massachusetts (2015–2017) designed to test a novel conceptual framework called Stakeholder-driven Community Diffusion. For whole-of-community interventions, this framework posits that diffusion of stakeholders’ knowledge about and engagement with childhood obesity prevention efforts through their social networks will improve the implementation of health-promoting policy and practice changes intended to reduce obesity risk. SUU5 used systems science methods (agent-based modeling, group model building, social network analysis) to design, facilitate, and evaluate the work of 16 multisector stakeholders (‘the Committee’). In this paper, we describe the design and methods of SUU5 using the conceptual framework: the approach to data collection, and methods and rationale for study inputs, activities and evaluation, which together may further our understanding of the hypothesized processes within Stakeholder-driven Community Diffusion. We also present a generalizable conceptual framework for addressing childhood obesity and similar complex public health issues through whole-of-community interventions.

## Introduction

Prevalence of obesity in early childhood is high and remains a national and global public health concern [1]. Obesity disproportionately affects racial and ethnic minorities, and contributes to lifelong health inequities that plague the most vulnerable populations [2–6]. Underlying causes of early childhood obesity are complex, multi-level, and interconnected. Making progress on addressing determining factors, both proximal (e.g.: food behaviors) and distal (e.g.: food environments), requires multifaceted intervention strategies, which is echoed by recent calls to integrate complex systems thinking into obesity prevention [7–12].

Whole-of-community childhood obesity prevention interventions are a promising approach in reducing population-level excess weight gain among children [13, 14]. Recent advances in whole-of-community interventions seek to integrate systems thinking in the implementation of community-wide practices and policies intended to promote healthy weights and reduce obesity risk. Further, whole-of-community interventions often engage groups of multisector stakeholders (e.g.: coalitions, task forces, advisory boards, steering committees) to facilitate intervention design, uptake and tailoring of strategies to local contexts. While previously completed whole-of-community interventions have made general observations about the importance of leadership engagement, they have not adequately explored group structure, activities, or characteristics, nor studied factors hypothesized to be critical to their success, such as implementation processes, and individual- and group-level dynamics [15].

Shape Up Under 5 (SUU5) was a pilot whole-of-community intervention that convened a 16-member stakeholder committee around the issue of early childhood obesity prevention in Somerville, Massachusetts between 2015–2017. It was part of the Childhood Obesity Modeling for Prevention and Community Transformation (COMPACT) Study: an international collaboration applying systems science approaches to understand optimal conditions for successful whole-of-community interventions (www.compactstudy.org). SUU5 builds on an observation which consistently emerged from several previous whole-of-community interventions: that the form and function of leadership groups is a critical and understudied aspect of intervention success [15]. SUU5 was designed to better understand how and why stakeholder groups succeed, and the conditions under which they create community-wide change.

SUU5 draws on systems science in the study design, implementation, and evaluation. It considers interactions among stakeholders from multiple sectors (known as heterogeneous actors), promotes understanding of community dynamics around addressing health behaviors, and recognizes nonlinear causes and effects of early childhood obesity [16, 17] [18, 19]. Data collection and evaluation methods identify and elucidate complex social interactions between community members, and potential unanticipated causes and results of changes within the system. Taken together these methods result in a novel, systems-based approach to studying and implementing community-based obesity prevention interventions. [12, 16, 17, 20].

This paper describes the SUU5 design, methodology, and conceptual framework, including study activities and data collection. We explain how those process and evaluation methods contribute to a greater understanding of Stakeholder-driven Community Diffusion, and how the work may lead to a better comprehension of diffusion mechanisms to impact community-wide systems change to prevent obesity in early childhood. Further, we consider replication and scale of the framework in other settings, including implications of using study data in real time versus for evaluative purposes only.

## Stakeholder-driven Community Diffusion conceptual framework

**Fig 1:**
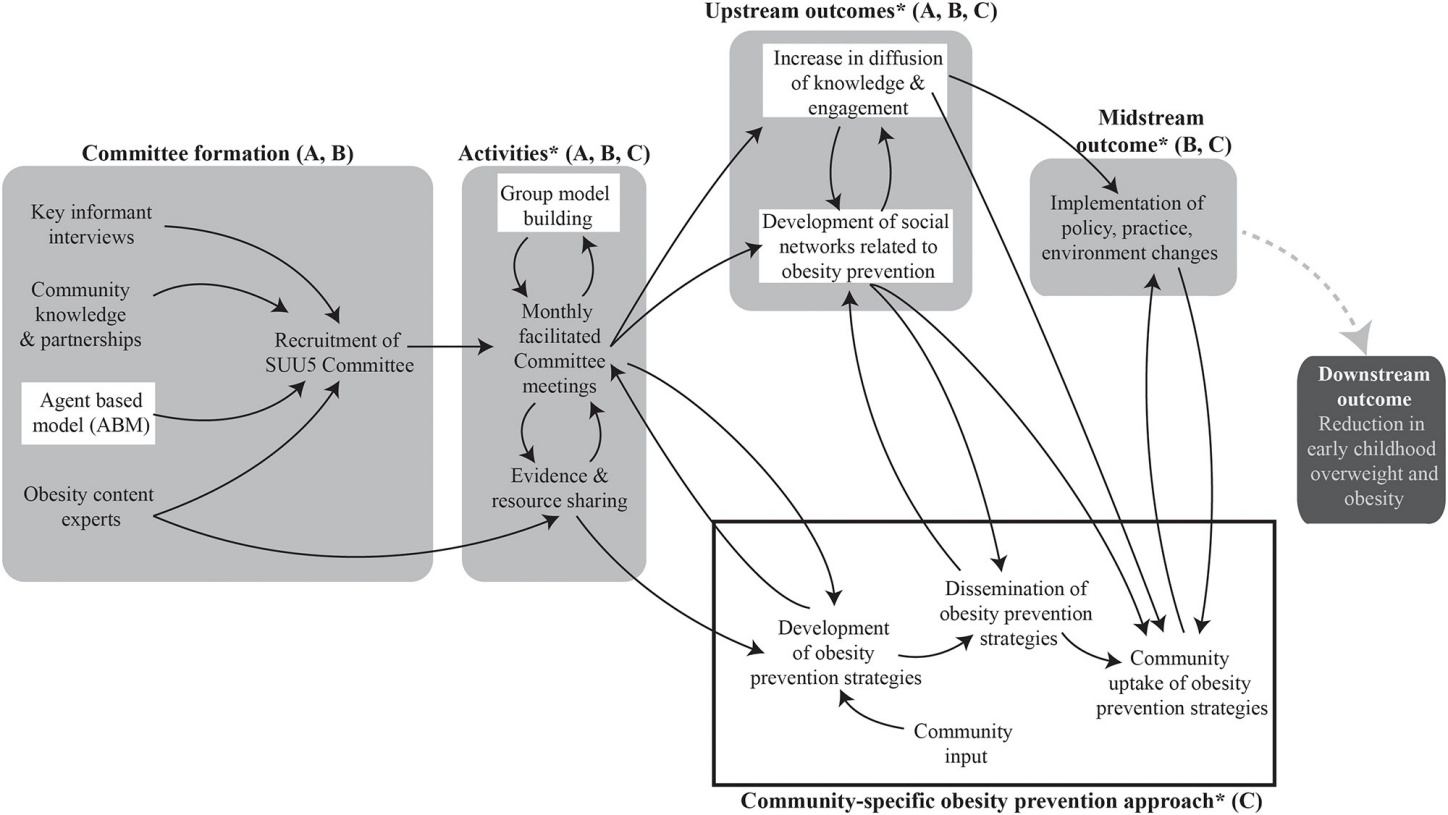
Stakeholder-driven Community Diffusion conceptual framework. Notes: (A) *White box indicates use of systems science; (B) Light gray shaded boxes indicate* **a priori** *conceptual framework; (C)* * *indicates data collection points*.

Fig 1 depicts the SUU5 conceptual framework, including stakeholder group (‘Committee’) formation, study activities, and outcomes. Stakeholder-driven Community Diffusion is the underlying theoretical mechanism driving SUU5, which posits that “upstream” diffusion of stakeholders’ knowledge about and engagement with childhood obesity prevention efforts throughout their social networks engenders “midstream” health-promoting policy and practice changes. These changes, in turn, may ultimately lead to“downstream” behavior change, and reductions in overweight and obesity prevalence among young children. The framework draws on theories from multiple disciplines, and is informed by the study of previously completed and successful obesity prevention trials. It proposes that individual attributes (i.e.: knowledge about and engagement with obesity prevention efforts) of those who design and implement whole-of-community interventions (i.e.: stakeholders) are important to an intervention’s success [13, 15, 21]. The diffusion science literature suggests that these salient persional attributes can change over time and subsequently be diffused throughout stakeholders’ social networks into the broader community, and create conditions that maximize policy and environmental change [22–24]. Additionally, the Stakeholder-driven Community Diffusion framework conceptual framework draws on key principles from (1) Diffusion of Innovations theory, which emphasizes the importance of intervention, stakeholder, and environmental attributes in stimulating the diffusion of an idea or intervention [25, 26], and (2) adult learning theories like Transformative Learning, that consider how individuals change their understanding over time [27].

Fig 1 highlights aspects of the study that use systems science methods, as well as the development and diffusion of a community-specific obesity prevention approach that the Committee developed over the two-year study period. While the four boxes shaded in gray were planned *a priori*, the specific obesity prevention approach (outlined in the black box) developed out of the Committee’s work together. Efforts to diffuse and evaluate the approach were both iterative and community-specific. Due to Committee’s multisectoral representation and desire for community wide impact, their prevention approach included several different targeted strategies, reaching different community members, at varying levels and in many sectors.

This conceptual framework allows for adaptation during all study phases, blending rigorous methodology with community co-creation, and supporting sustained community-wide impact. Further, the community-specific obesity prevention approach can incorporate several targeted strategies at once, ensuring that efforts are seen in many places and levels at one time. Due to the pilot nature of the work we did not measure the furthest downstream outcome of child weight, shown in the dark gray shaded box.

### The importance of knowledge, engagement, and social networks

At the core of Stakeholder-driven Community Diffusion are the dynamic connections and relationship structures between stakeholders, through which their personal attributes can change and are diffused. Specifically, each stakeholder has a varying level of knowledge and engagement related to early childhood obesity prevention, which is hypothesized to determine their willingness and ability to impact prevention efforts at different time points [21]. In this context, knowledge is defined as the understanding an individual has about community-wide efforts to prevent childhood obesity; engagement is a latent construct variable that captures the passion, motivation, and stewardship an individual has for preventing early childhood obesity. Diffusion of knowledge and engagement may be bidirectional between stakeholders, and will be influenced by the network structure; changing levels of knowledge and engagement among all stakeholders in the network will impact the environments they inhabit.

The *knowledge* construct is comprised of five domains reflecting an individual’s understanding of areas related to early childhood obesity: (1) the problem, (2) modifiable intervention factors, (3) other people and roles involved in prevention, (4) how to intervene sustainably, and (5) available resources to promote prevention efforts. These five facets were drawn from an extensive review of both successfully completed obesity prevention interventions, and other relavent survey instruments [21]. The *engagement* construct is made up of five domains measuring different aspects of an individual’s enthusiasm and agency for preventing childhood obesity: (1) dialogue and mutual learning, (2) flexibility regarding prevention efforts, (3) influence over others and in the community, (4) leadership capacity, (5) and trust in others. *Social networks* are personal and professional relationships related to childhood obesity prevention efforts. These represent the number, direction, and structure of ties that a person has related to such work, and the pathways through which knowledge and engagement on the topic are diffused. There are three qualities that describe the strength of each relationship: closeness, frequency of interaction, and level of influence.

## Committee formation, study activities, and committee outputs

The research team convened a 16-member stakeholder Committee of early childhood professionals from six sectors in Somerville, MA: early education and care, parks and recreation, the local health department, healthcare, food assistance programs, and the Somerville Public Schools. Committee members were recruited and selected based on four inputs: key informant interviews, community knowledge and partnerships, obesity content expert reccommendations, and an agent-based model populated with data collected from participants involved in the Shape Up Somerville intervention (2003–2005) [28].

The Committee was tasked with discussing and addressing the issue of early childhood obesity during 16 Committee meetings held every four to six weeks over the course of the two-year study period (October 2015 to October 2017). Meetings were planned with input from experts in Group Model Building: a participatory-research method through which a small group addresses a complex problem with foundations in systems dynamics [29].

Through the meeting process, among several emergent themes, the Committee identified a community-wide need for a set of consistent messages about the importance of healthy growth in young children, and recognized the importance of promoting culturally-appropriate and relatable strategies for adopting obesity-preventing behaviors. Ultimately this became a messaging effort entitled *Small Steps*: *Eat Play Sleep*. This campaign was built on four evidence-based practices for children from birth to age five: eating well, playing actively, limiting screen time, and developing good sleep habits. The campaign includes a poster, set of age-appropriate brochures with tips and resources for healthy growth, introductory and training videos for families and providers, and a set of discussion points for conversing with families and caregivers about the importance of healthy habits. All campaign materials are currently maintained by the City of Somerville, available for free download and use in four languages: English, Spanish, Portuguese, and Haitian Creole (somervillehub.org/downloads).

All study procedures and activities were approved by the Institutional Review Board at Tufts University, including creation of partnerships and data sharing with COMPACT researchers and consultants from other universities and institutions. We obtained informed consent for all study activities from all study participants, which included SUU5 Committee members and 1^st^ degree alters, the individuals that each Committee member named into their social networks. Committee members provided written informed consent for participation in meetings, surveys, and interviews. 1^st^ degree alters provided electronic consent for participation in online surveys only, and to indicate if they were willing to participate in a semi-structured interview related to their perceptions of the *Small Steps*: *Eat Play Sleep* campaign.

## Evaluation approach and rationale

Shape Up Under 5 used mixed methods data collection to evaluate the diffusion of knowledge and engagement among stakeholders’ social networks, and explore the link between the up- and midstream outcomes described in the conceptual framework. Table 1 provides an overview of the data collection methods during each stage, and Table 2 shows the data collection timeline.

**Table 1:** Shape Up Under 5 data collection methods and rationale.

Conceptual framework area	Measure	Participants	Method	Description
**Activities**	Committee meetings	Committee meeting attendees	Detailed meeting minutes taken by research staff	Thoughts and themes expressed by the SUU5 Committee members and research team during monthly meetings, subcommittee meetings, and makeup meetings
	Group model building visualizations	Committee meeting attendees	Scripts and participatory activities	Systems and connections related to early childhood obesity prevention in Somerville
	Mini COMPACT Stakeholder-Driven Community Diffusion exit surveys	SUU5 Committee members	Paper survey	Knowledge, engagement, and social network change as a result of SUU5 meeting attendance
**Upstream outcomes**	***COMPACT Stakeholder-driven Community Diffusion survey***			
	Knowledge (K)	SUU5 Committee members, 1^st^ degree alters	Online survey	Five areas related understanding of early childhood obesity prevention efforts: the problem, modifiable intervention factors, roles involved in prevention, sustainability, and available resources
	Engagement (E)	SUU5 Committee members, 1^st^ degree alters	Online survey	Five areas related to passion and motivation to address early childhood obesity: dialogue, flexibility, influence, leadership capacity, and trust in others
	Social networks (N)	SUU5 Committee members, 1^st^ degree alters	Online survey	Relationship structure and characteristics (closeness, influence, frequency of interaction)
	Transformative learning	SUU5 Committee members	Online survey Semi-structured qualitative interview	Perspective shifts and integration of new information on the topic of early childhood obesity efforts
**Midstream outcomes**	Policies, practices, and environments related to obesity prevention	SUU5 Committee members	Online survey	Assessment of the community and built environment, staff training and client outreach, and implementation of best policies and practices in childcare settings
**Community-specific obesity prevention approach (*Small Steps*: *Eat Play Sleep* campaign)**	Campaign distribution, exposure and reach	SUU5 Committee members 1^st^ degree alters	Paper measurement logs Online survey	Location and amount of campaign material distribution; description of exposed population Details of exposure to campaign
	Campaign dissemination	SUU5 Committee members	Semi-structured qualitative interview	Location and amount of distribution of campaign materials; description of exposed population by Committee members
	Campaign perceptions	1^st^ degree alters	Semi-structured qualitative interview	Perception of Small Steps: Eat Play Sleep campaign among community members

**Table 2:** Shape Up Under 5 data collection timeline.

*Year*	2015			2016												2017							
*Month*	10	11	12	1	2	3	4	5	6	7	8	9	10	11	12	1	2	3	4	5	6	10	11
**Activities–SUU5 Committee**																							
Committee meetings	√		√	√	√		√	√	√	√		√	√	√		√	√	√		√	√		
Mini COMPACT Stakeholder-Driven Community Diffusion exit surveys			√	√	√		√	√	√	√		√	√	√		√	√	√		√	√		
Group Model Building activities and output	√		√	√			√			√			√	√			√						
**Upstream outcomes**																							
COMPACT Stakeholder- Driven Community Diffusion survey																							
*SUU5 Committee K&E*	√						√							√						√		√	
*SUU5 Committee N*	√			√			√			√				√			√			√		√	
*SUU5 Committee first degree alters KEN*		√						√							√						√		√
SUU5 Committee semi-structured qualitative interview	√													√									√
**Midstream outcomes**																							
Policy, Practice, Environment survey	√													√								√	
**Community-specific obesity prevention strategy**																							
Measurement logs*																		√	√	√	√	√	√
Small Steps: Eat Play Sleep online survey*																				√			√
Small Steps: Eat Play Sleep Interviews*																				√		√	√

Notes:

K: **K**nowledge

E: **E**ngagement

N: social **N**etworks

*Indicates data collection methods incorporated to measure the community specific obesity prevention strategy

### Upstream outcomes—Knowledge, engagement, social networks

We developed the multi-method COMPACT Stakeholder-driven Community Diffusion Survey to measure social networks, knowledge, and engagement [21]. The web-based survey was distributed semi-annually to two groups of respondents: SUU5 Committee members, and individuals named into Committee members’ social networks (i.e.: the Committee members’ first degree alters). Full text of the web-based survey is available as an appendix in a related manuscript [21]. The social network module of the survey was administered an additional two times per year with the SUU5 Committee members only, in an attempt to capture more frequent changes in networks over time. Additionally, we collected mini COMPACT Stakeholder-driven Community Diffusion “exit” surveys after each Committee meeting from all SUU5 Committee members in attendance at the meeting. These one-page paper surveys were completed anonymously after each meeting, and captured information about each person’s knowledge of the specific meeting topic, passion and enthusiasm for taking action steps related to the meeting topic, and new connections with committee members as a result of attending the meeting.

To complement electronic and paper survey data, we conducted three annual semi-structured qualitative interviews with each Committee member. These interviews provided additional insights on members’ experiences participating in SUU5. Questions varied slightly in each round but were designed to deepen our understanding of the impact of Committee participation on the study participants, and to elucidate which parts of the Committee’s process contributed to the group’s success. Additionally, the interviews focused on the process of Transformative Learning to assess whether and how Committee members integrated new information, perspectives, and practices into their work related to early childhood obesity prevention, as a result of Committee participation [27]. A sample interview guide may be provided to interested readers upon request to the corresponding author.

### Midstream outcomes—Policies, practices, and environments

To assess the impact of the Committee’s work on the midstream outcomes (i.e.: environmental and policy change), we utilized a survey to measure policies, practices, and environments related to early childhood obesity prevention efforts in Somerville. The survey included three modules to capture (A) the community and built environment (5 items), (B) staff training and family outreach (9 items), and (C) implementation of best practices and policies for childcare providers (3 items). We adapted items from existing policy and environmental assessments, including the Nutrition and Physical Activity Self-Assessment for Child Care survey [30], the Rudd Center’s Child Care Nutrition and Physical Activity Assessment Survey [31], the Let’s Move Childcare Checklist [32], and also created new items. The web-based survey was distributed annually. All Committee members received module A; direct services providers (e.g. pediatricians, home visitors) received modules A and B; and childcare providers received all three modules. Full text of the survey may be requested from the corresponding author, in an effort to track the instrument’s distribution and use by other investigators.

### Community-specific obesity prevention approach

We developed evaluation strategies to measure diffusion and community uptake of the *Small Steps*: *Eat Play Sleep* messaging campaign with input and feedback from the Committee to ensure that materials were easy to use and captured relevant information. Committee members used measurement logs to document the type and amount of materials distributed and general descriptions of the exposed populations. Individuals nominated to Committee members’ social networks were invited to complete a web-based survey about whether they had been exposed to the campaign, and how they perceived it. We also conducted interviews with Committee members and members of their social networks (i.e.: 1^st^ degree alters) to gauge the dissemination and community uptake of the campaign materials.

### Approach to data collection

The varying types of data collection were designed to prospectively test the Stakeholder-driven Community Diffusion framework in several ways. Data from the COMPACT Stakeholder-driven Community Diffusion survey will populate an agent-based model that simulates changes in knowledge among community members (the “agents”) through repeated social interactions. We also used an agent-based model *a priori*, to provide insight into how committee composition might influence trends in knowledge and engagement throughout the community.

Evaluation of policy, practice, and environment change and the messaging campaign will provide a link between the Committee’s work and change at the community-level supporting obesity prevention efforts. Supplemental interviews with Committee members support and expand upon the quantitative data collection, further explaining the link between Committee participation and changed perspectives, and action steps for early childhood obesity prevention.

## Applying and scaling the conceptual framework

One strength of our framework is that it fuses the conceptual (i.e.: Stakeholder-driven Community Diffusion framework) with the contextual (i.e. community-specific obesity prevention mechanism). The rigorous methodological underpinnings of the framework integrate best practices and learnings from several disciplines in systems science–agent-based modeling, group model building, and social network analysis–as well as obesity science, and participatory community engagement. The context-specific obesity prevention approach, exemplified in this case by the *Small Steps*: *Eat Play Sleep* campaign, was initiated from a thorough and community-driven process that recognized the unique needs of the target population. This blending of conceptual and contextual represents a critical step in our understanding of what affects adoption, fidelity, and sustainability of a community-based intervention. Further, this framework may be adapted into other settings, and applied to similarly complex, persistent, and systemic public health issues.

### Considerations and implications for future work

One challenge we faced in applying this conceptual framework was finding the balance between the fidelity of data collection necessary for rigorous hypothesis testing, with monitoring efforts and use of data in real-time to inform intervention efforts and community action. We evaluated upstream outcome data on knowledge, engagement, and social networks only *post hoc*, drawing connections between upstream and midstream outcomes only after the study activities were completed. This tension between data collection for hypothesis testing, and for immediate use may be inherent in community-based interventions that are scaffolded by a rigorous methodological framework. While use of systems science intervention techniques and measurements in this context, in particular that of agent-based modeling, Group Model Buidling and social network analysis, may help to alleviate some of this inherent tension, more research on this topic is needed.

Future work could explore how those constructs (i.e.: knowledge, engagement, and social networks) and the Stakeholder-driven Community Diffusion conceptual framework, can be systematically measured, and efficiently and thoughtfully deployed in real time to better execute community-based interventions as they happen. Also, while this study highlighted the connection between up- and midstream outcomes, additional work should look to corroborate the longer term linkages to the downstream outcome: reduction in early childhood overweight and obesity.
